# Secondary Sutureless Posterior Chamber Lens Implantation with Two Specifically Designed IOLs: Iris Claw Lens versus Sutureless Trans-Scleral Plugs Fixated Lens

**DOI:** 10.3390/jcm10102216

**Published:** 2021-05-20

**Authors:** Daniel Seknazi, Donato Colantuono, Rachid Tahiri, Francesca Amoroso, Alexandra Miere, Eric H. Souied

**Affiliations:** 1 Department of Ophthalmology, Centre Hospitalier Intercommunal de Créteil, 94010 Créteil, France; daniel.seknazi@gmail.com (D.S.); amorosofrancesca123@gmail.com (F.A.); alexandra.miere@chicreteil.fr (A.M.); eric.souied@chicreteil.fr (E.H.S.); 2 Granville Center of Ophthalmology, 50400 Granville, France; tjhr78@hotmail.com

**Keywords:** secondary implantation, sutureless fixation, sutureless trans-scleral plugs fixated lens, iris claw lens

## Abstract

Background. The management of patients with aphakia and/or lack of capsular support remains debated. The sutureless posterior chamber IOL (PCIOL) fixation is a very useful surgical option. The purpose of the study was to compare the early outcomes as well as post-operative best corrected visual acuity, refractive errors and complications of two different techniques of sutureless PCIOL secondary implantation. Methods. Patients who underwent secondary implantation from December 2019 to January 2021 in the Department of Ophthalmology of Creteil Hospital, and in the Granville Ophthalmology Center, were retrospectively included. Eyes implanted with the iris claw lens (Artisan Aphakia IOL model 205, Ophtec BV, Groningen, The Netherlands) were included in group 1, and eyes implanted with a newly developed sutureless trans-scleral plugs fixated lens (STSPFL, Carlevale lens, Soleko, Pontecorvo, Italy) were included in group 2. Results. Twenty-two eyes of 22 patients were enrolled in group 1, and twenty eyes of 20 patients in group 2. No difference was found in visual acuity between two groups (0.35 +/− 0.29 logmar for group 1 and 0.23 +/− 0.51 logmar for group 2) (*p* = 0.15) at mean post-operative follow up (6.19 +/− 3.44 months for group 1 and 6.42 +/− 3.96 months for group 2) (*p* = 0.13). Both the mean refractive error (MRE) and induced astigmatism (IA) were greater in group 1 compared to group 2, respectively: the MRE was 0.99 +/− 0.57 vs. 0.46 +/− 0.36 (*p* < 0.01), and IA was 1.72 +/− 0.96 vs. 0.72 +/− 0.52 (*p* < 0.01). Conclusions. No significant differences in terms of the recovery of visual acuity were found between the two groups. Group 2 (STPFL) gives better results in our sample due to less post-operative induced astigmatism and less refractive error.

## 1. Introduction

The surgical solution for patients with aphakia and/or lack of capsular support remains a debated topic. Currently, the available options are the implantation of the intraocular lens (IOL) in the anterior chamber (ACIOL) or the fixation of the IOL to the iris or the sclera, in the posterior chamber (PCIOL) [1]. The placement of the ACIOLs in the iridocorneal angle is relatively simple, but it requires a large corneal or scleral incision and is often associated with complications, such as important induced astigmatism (IA), transient corneal edema, bullous keratopathy, increased intraocular pressure (IOP), uveitis or hyphema [2]. PCIOLs can be sutured directly to the iris using non-absorbable sutures. Although this procedure is very useful, especially in the case of the dislocation of the entire IOL-capsular bag complex, complications associated with sutures and iris damage are very frequent [1].

Indeed, the implantation of the iris claw lens has become a surgical option that is increasingly adopted by surgeons [3]. According to the classic procedure, the specifically designed IOL is injected through a 5.5 mm incision and anchored posterior to the iris by fine haptics, without sutures, with more stability and lower risk of iris injury [3]. This technique has very good visual outcomes and low complication rates, even though the large corneal incision induces significant postoperative astigmatism and the integrity of the iris diaphragm is an indispensable requirement for the surgery [1]. Conversely, other PCIOLs, such as sutured sclera-fixated IOLs (SSF-IOL), do not require neither iris nor capsular support and can also be applied in the case of dislocation of the entire capsular bag, even if the classic procedure is technically demanding. The suture is used to place the haptics of the IOL through the pars plana or the sulcus, and the needle for scleral fixation can be passed ab externo (from outside of the eye) or ab interno (from inside of the eye) [4]. Sutures may cause complications. such as inflammation, suture knot exposure, suture breakage, pseudophakodonesis, IOL subluxation, intraocular hemorrhage and even sutured-related endophthalmitis [4]. For this reason, suture-free scleral fixation techniques have gained popularity in recent years. After the ancillary description provided by Maggi and Maggi [5], Gabor et al. [6] introduced sutureless fixation of the IOL by making scleral tunnels parallel to the limbus with a 24-gauge needle, in which the haptics were then incarcerated. Afterwards, many surgeons revised the technique, trying to improve the fixation and the stability of the lens [1,2,7,8,9,10]. Nevertheless, among the principal limitations of these procedures was that the lenses used for intrascleral placement were standard three-piece IOLs, which were not designed to be placed in the sclera. Recently, a folding acrylic, sutureless trans-scleral plugs fixated lens (STSPFL, Carlevale intraocular lens, Soleko SPA, Pontecorvo, Italy) has been introduced with excellent results [11]. It is specifically realized for scleral sutureless fixation, and it is equipped with a small plug attached to each of two haptics to allow the anchorage of the lens under the created scleral flap with a self-blocking mechanism. In this study, we aimed to compare the techniques of secondary implantation with either the iris claw IOL or sutureless trans-scleral plug fixated lens, to evaluate the early outcomes as well as post-operative best corrected visual acuity, refractive errors and complications of the two different sutureless PCIOL fixation procedures. To our knowledge, no comparative studies have been published regarding the two mentioned PCIOLs.

## 2. Materials and Methods

### 2.1. Inclusion Criteria and Study Design

Consecutive patients with aphakia or IOL-capsular bag luxation/subluxation and treated with the two different techniques of PCIOL implantation, presenting at the Department of Ophthalmology of the Hospital Intercommunal Center of Creteil and Granville Ophthalmology Center, between December 2019 and January 2021, were retrospectively considered for inclusion. All included patients were evaluated at baseline, before the secondary implantation surgery and at 6 months of follow up. The preexistence of other ophthalmological pathologies was noted and did not represent a criterion of exclusion for the patients. In patients with aphakia after complicated cataract surgery, the secondary implantation was performed with a second surgery. This retrospective study was performed in agreement with the Declaration of Helsinki for research involving human subjects. This study had the approval of the Ethics Committee of the Federation France Macula 2020-153 and was carried out in compliance with French legislation. Two different surgeons (D.S. and R.T.) with equivalent skills performed the surgery. According to the type of IOL used, patients were divided into two groups. Group 1 was implanted with the iris claw IOL (Opthtec, Artisan Aphakia model 205), and group 2 was implanted with STSPFL (Carlevale intraocular lens, Soleko SPA, Pontecorvo, Italy). Post-operative best corrected visual acuity was obtained using ETDRS and converted in logMAR. Post-operative refractive error, defined as the difference between the spherical equivalent and the expected value resulting from biometry, was evaluated. Post-operative complications, including macular edema, IOL displacement, vitreous bleeding, hypotony, conjunctival erosion and retinal detachment were evaluated. Spectral-domain OCT (Spectralis HRA + OCT; Heidelberg Engineering, Germany) examination was performed at the follow-up visit. The detection of any intraretinal cystoid spaces was registered as post-operative macular edema. The lens displacement was considered when the IOL edge involved the visual axis causing a significant loss of vision.

### 2.2. Intraocular Lenses

#### 2.2.1. Iris Claw IOL

**Figure 1:**
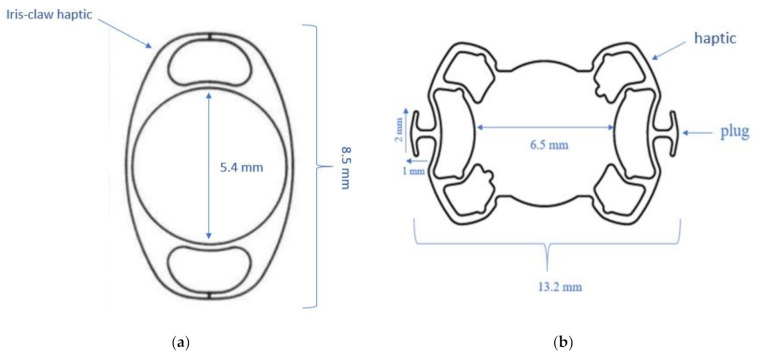
(**a**) Schematic drawing of an iris claw lens (Artisan, Ophtec). (**b**) Schematic drawing of a sutureless trans-scleral plugs fixated lens (Carlevale lens, Soleko).

The Artisan lens (Artisan aphakia model 205, Ophtec, Boca Raton, FL, USA) (Figure 1a) is a polymethylmethacrylate (PMMA) lens with a total length of 8.5 mm and a central 5 mm optic supported by two unique flexible haptic “claws” for iris fixation.

#### 2.2.2. Sutureless Trans-Scleral Plugs Fixated Lens

STSPFL (Carlevale lens, Soleko SPA, Pontecorvo, Italy) (Figure 1b) is a uniquely designed, foldable, acrylic IOL with 25% water content and a UV filter. It has an optical diameter of 6.5 mm and a total diameter of 13.2 mm. The haptic angulation is of 10°. Each of them is equipped with a plug (width of 2 mm and length of 1 mm) attached almost perpendicularly to the haptic long side. The IOL refractive index is 1.461, and the recommended injector system is Medicel Viscojet 2.2 or 2.7.

### 2.3. Surgical Procedure

All patients underwent vitrectomy and/or completion of vitrectomy before IOL implantation; lens removal with a vitreous cutter and explant of dislocated IOL were performed as combined procedures, when required. The surgery was performed with either general anesthesia (AG) or retrobulbar anesthesia (AR) for the two groups. The choice of anesthesia was agreed by the surgeons, the anesthetist and the patient, evaluating the patient’s comorbidities and preferences. Precisely, in the iris claw group, 10 patients underwent AG and 12 AR, while in group 2, nine underwent AG and 11 AR. Phenylephrine chlorhydrate 5.4 mg + tropicamide 0.28 mg insert opht (Myriasert, Thea Pharmaceuticals Ltd., Clemont-Ferrand, France) was always used for pupil dilatation before surgery.

#### 2.3.1. Iris Claw Group (Group 1)

The iris claw implantation was realized using a standardized technique: creation of two horizontal small corneal incisions at 3 and 9 o’clock; preparation of a 6 mm corneal incision at 12 o’clock; injection of cohesive viscoelastic into the anterior chamber; insertion of the IOL (Artisan aphakia model 205, Ophtec, Boca Raton, FL, USA) through the corneal incision; rotation of the lens to allow the haptic orientation at 3 and 9 o’clock; manipulation of the lens with iris claw-holding forceps through the corneal incision to reach the pupil area behind the iris plane; enclavation of the midperipheral iris between the claw haptics with a small spatula by applying gentle pressure; suture of the corneal incisions with 10-0 nylon suture; and washing of the viscoelastic with a bimanual irrigation/aspiration system (Constellation Vision System; Alcon Laboratories, Inc., Fort Worth, TX, USA).

#### 2.3.2. Sutureless Trans-Scleral Plugs Fixated Lens Group (Group 2)

**Figure 2:**
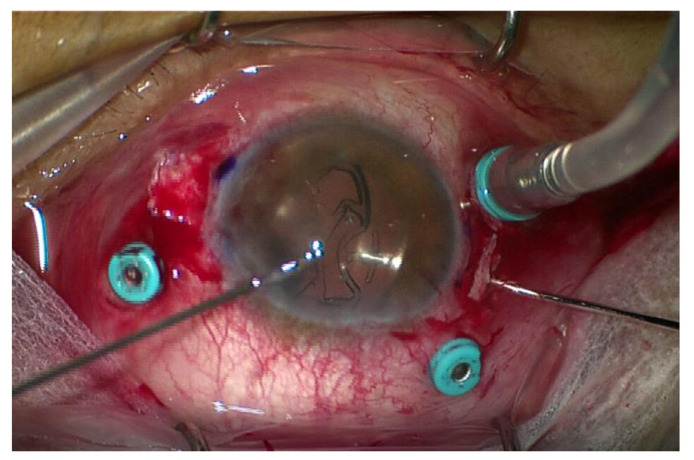
Carlevale lens fixation: the 25-gauge forceps are passed through the side anterior chamber incision to maintain the second haptic of the IOL and to gently move it under the iris, so that the second plug is gripped by another pair of 25-gauge forceps and passed through the opposite sclerotomy.

The technique of implantation of the STSPFL (Supplementary Materials Video S1)included the following stages: conjunctival peritomy; realization of two partial-thickness limbal-based scleral flaps (about 4.0 × 4.0 mm) lined up 180° from each other; creation of 2 sclerotomies using a 25-gauge needle at 2.0 mm from the limbus in correspondence to the 0° to 180° axis; injection of the STSPFL into the anterior chamber through the 2.2 mm corneal tunnel and grasping of the leading plug by crocodile tip forceps (Grieshaber Maxgrip Forceps 25G) inserted into the vitreous chamber through the sclerotomy and externalization of the plug under the scleral flap in a single maneuver; grasping of the trailing plug and externalization with 2 forceps using the handshake technique; and achievement of IOL centration without extra intraoperative maneuvers (Figure 2).

The sealing of scleral flaps and the conjunctival wound with polyglactin 8–0 Vicryl suture and the closure of the corneal incision with hydrosuture was realized.

### 2.4. Statistical Analysis

Statistical analysis was performed using STATA version 13.0 (Texas, TX, USA). Qualitative variables were described in percentages, and quantitative variables were described as mean ± standard deviation. The Wilcoxon–Mann–Whitney test was used to compare measured parameters. *p* < 0.05 was considered significant.

## 3. Results

A total of 42 eyes of 42 patients were retrospectively included, 22 eyes in group 1 and 20 in group 2. The mean age was 76.3 +/− 10.3 years in group 1 and 72.9 +/− 8.7 years in group 2 (*p* = 0.24). The axial length was 23.8 +/− 2.3 mm in group 1 and 24.1 +/− 1.5 mm in group 2, with no significant difference between the two groups (*p* = 0.12). Of the included eyes in group 1, 11/22 (50.0%) presented posterior capsule rupture, while the remaining 11/22 (50.0%) presented dislocated IOL. In group 2, 12 out of 20 eyes (60.0%) presented posterior capsule rupture, and 8 out of 20 (40.0%) presented dislocated IOL. In group 1, 15 eyes had no previous comorbidities, while seven (31.82%) eyes presented preexisting comorbidities consisting of: two eyes with age-related macular degeneration (AMD) (9.09%), two eyes with myopic maculopathy (9.09%), two eyes with retinal detachment (9.09%) and one eye with macular hole (4.54%). In group 2, 14 eyes had no previous comorbidity and six eyes (30.0%) presented preexisting comorbidities: two eyes (10.0%) had age related macular degeneration (AMD), one eye (5.0%) had myopic maculopathy, one eye (5.0%) was treated for Irvine-Gass syndrome, one eye (5.0%) had diabetic macular edema and one eye (5.0%) presented a corneal scar. The clinical characteristics of the two groups are reported in Table 1.

The mean follow-up was 6.19 +/− 3.44 months for group 1 and 6.42 +/− 3.96 months for group 2. (*p* = 0.13). The mean post-operative best-corrected visual acuity (BVCA) after surgery at follow up was 0.35 +/− 0.29 logMAR in group 1 and 0.23 +/− 0.51 in group 2 (*p* = 0.19). Among all 42 included eyes, 29/42 had no pre-existing comorbidities (69.05%). In this subgroup of eyes, the mean BVCA was 0.28 +/− 0.26 logMAR in group 1 and 0.14 +/− 0.11 logMAR in group 2 (*p* = 0.06). The mean refractive error after surgery was 0.99 +/− 0.57 D in group 1 and 0.46 +/− 0.36 D in group 2 (*p* < 0.01). The mean induced astigmatism was 1.72 +/− 0.96 D in group 1 and 0.72 +/− 0.52 D in group 2 (*p* = 0.01). The comparison of the main post-operative outcomes between the two groups is shown in Table 2.

Concerning the post-operative complications in group 1, two eyes (9.09%) had IOL dislocation, and three eyes (13.64%) presented cystoid macular edema: two of them regressed after medical treatment with corticoids and anti-inflammatory eye drops, and one was refractory to medical treatment and was present at the last follow up. Furthermore, one eye (4.54%) underwent massive vitreous hemorrhage that required a new surgical procedure, and one eye (4.54%) presented self-limiting hyphema (Table 3).

In group 2, two eyes (10.0%) had cystoid macular edema: one of them regressed after medical treatment with corticoids and anti-inflammatory eye drops, and one required supplemental retrobulbar corticosteroids injection for complete resolution. Furthermore, one eye (5.0%) had mild vitreous hemorrhage which regressed spontaneously after 1 month of follow up, one eye (5.0%) had neurotrophic ulcer that resolved after treatment with lubricating eye drops and one more eye (5.0%) had a broken plug during surgery, needing immediate IOL removal and the implantation of a new lens of the same model.

## 4. Discussion

In this study, we compared the early outcomes of eyes undergoing iris claw IOL and STSPFL fixation as a surgical solution for secondary implantation. The use of a PCIOL offers many advantages: it takes place near the original lens position; it is distant from corneal endothelium and angle structures; it provides a good mechanical barrier between the vitreous cavity and anterior chamber [1]. Among the PCIOLs, the iris claw lens is universally considered as a very reliable surgical option, while very recently, the use of sutureless trans-scleral plugs fixated lenses is spreading among surgeons [12,13]. In our study, the visual outcomes in the iris claw group (group 1) were comparable with the STSPFL group (group 2) both in the overall cohort of patients and in the subgroup without comorbidities (*p* = 0.15 and *p* = 0.08, respectively). Our results are consistent with other studies in the literature reporting the early efficacy of the two surgical techniques [12,13]. Indeed, Barca et al. [14] reported similar mean post-operative visual outcomes (0.13 ± 12 logMar) and refractive errors (0.71 ± 1.21) at 8 months of follow up in patients who underwent STSPFL implantation, also reporting the great stability of the lens without cases of IOL dislocation and pseudophacodonesis. Conversely, the same authors described two cases of reverse pupillary block with pigment dispersion probably related to excessive postoperative inflammation. In our series, we did not observe this complication, so we did not routinely perform peripheral iridectomy with the vitrector probe at the end of the surgical procedure as suggested by the authors. However, longer post-operative follow up is needed because the block can occur even years after the surgery.

**Figure 3:**
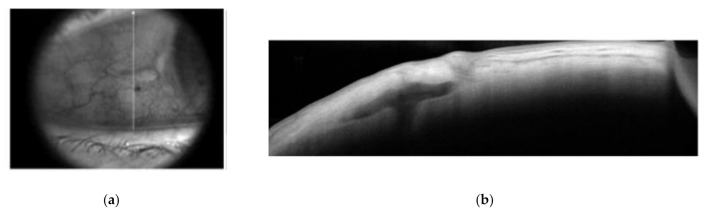
(**a**) Infrared image of the scleral flap in a patient implanted with the Carlevale IOL; (**b**) optical coherence tomography of the scleral flap showing the correct placement of the plug in the same patient.

Interestingly, in our series, the most important differences between the two groups were in terms of the mean induced astigmatism (1.72 +/− 0.96 vs. 0.72 +/− 0.52; *p* < 0.01) and mean refractive error (0.99 +/− 0.57 vs. 0.46 +/− 0.36; *p* < 0.01). This dissimilarity between the iris claw group and the STSPFL group may be explained by the difference in the size of the corneal incision, that is, of about 6 mm for the iris claw and 2.2 mm for the STSPFL, resulting in a more important post-operative induced astigmatism for the iris claw group (group 1). Conversely, in group 2, the symmetrical realization of scleral flaps 180° away and the anchorage of the plugs under the flaps allowed the natural centration and the great stability of the IOL, resulting in an excellent postoperative refractive outcome. Furthermore, the Carlevale lens was fixed without conjunctival erosion or inflammation (Figure 3a,b).

Additionally, regarding postoperative complications, in group 1, there were two cases of IOL dislocation, one case of vitreal hemorrhage and one case of hyphema. This may be a consequence of the stress that the iris claw implantation causes on the iris diaphragm, which, in some cases, may be damaged during the enclavation procedure, as already reported in the literature [15]. Bogumiła Sędziak-Marcinek et al. [16] recently reported the results of the secondary anterior-chamber implantation of iris claw on a large cohort of 132 patients, describing the procedure to be easier and shorter than posterior chamber iris claw lens fixation, to minimize the risk of postoperative macular edema, intraocular hemorrhagic complications and also retinal detachments with a mild reduction in the number of corneal endothelial cells. According to our experience, the placement of the iris claw in the more physiological retropupillar space may be easier in vitrectomized eyes with a very loose iris diaphragm; however, we did not analyze the endothelial cell count, and further studies should be performed to clarify this subject.

In group 2, the main complications were the break of the plug and the neurotrophic ulcer. Indeed during surgery for STSPFL implantation, the plugs are gripped by end-gripping 25-gauge vitrectomy forceps and passed through the sclerotomies. Therefore, in some cases, the procedure could be traumatic for the plugs, resulting in broken plugs and consequent IOL explantation. As for the neurotrophic ulcer, it may be related to performing two partial-thickness limbal-based scleral flaps at 3 and 9 o’clock, where it is indeed possible to intercept the passage of the ciliary nerves, leading to damage that may cause a loss of corneal sensibility and the neurotrophic ulcer. This may be avoided by performing the scleral flaps vertically (at 12 and 6 o’clock) and could also be very useful in the case of patients with large corneas. This study has several limitations, among which its retrospective nature, the small sample size and the short follow up. Given that our experience with STSPFL implantation is very recent, we believe it is important to provide the first results of comparison with another mainstream surgical technique for secondary implantation, such as iris claw implantation. To the best of our knowledge, this is the first study comparing complications and early outcomes between iris claw and STSPFL implantation.

In conclusion, no significant differences in terms of functional outcomes (i.e., BCVA) were found between the iris claw group and the STSPFL group. Nevertheless, the STSPFL group had better results due to less post-operative induced astigmatism and less refractive error. This technique might represent a useful surgical option for the management of aphakia, IOL–bag complex dislocation and lens subluxation, with great characteristics of stability. The advantages of this procedure are the absence of haptic manipulation, the self-centration and the firm blockage of the lens. Moreover, in patients with large corneas or to avoid the potential risk of neurotrophic ulcer, the vertical realization of scleral flaps (at 12 and 6 o’clock) could be routinely considered. Further studies, on larger cohorts and with longer follow up, are needed to confirm these preliminary results.

## Figures and Tables

**Table 1:** Demographic and clinical characteristics of study eyes.

	Group 1 (Iris Claw)	Group 2 (STSPFL)	Significance *
Patients/eyes	22	20	
Age (mean years +/− SD)	76.3 +/− 10.3	72.9 +/− 8.7	*p* = 0.24
Male	12	10	
Female	10	10	
Axial length (mm)	23.8 +/− 2.3	24.1 +/− 1.5	*p* = 0.12
Surgical indication			
Posterior capsule rupture	11 (50.0%)	12 (60.0%)	*p* = 0.55
Lens luxation	11 (50.0%)	8 (40.0%)	*p* = 0.55
Preexisting comorbidity	7 (31.82%)	6 (30.0%)	
AMD	2 (9.09%)	2 (10.0%)	
Myopic maculopathy	2 (9.09%)	1 (5.0%)	
Retinal detachment	2 (9.09%)	0	
Macular hole	1 (4.54%)	0	
Irvine-Gass syndrome	0	1 (5.0%)	
Corneal scare	0	1 (5.0%)	
Diabetic macular edema	0	1 (5.0%)	

* Mann–Whitney–Wilcoxon test.

**Table 2:** Comparison of main post-operative outcomes between two groups.

	Group 1 (Iris Claw) N = 22	Group 2 (STSPFL) N = 20	Significance *
Follow up (mean months +/− SD)	6.19 +/− 3.44	6.42 +/− 3.96	*p* = 0.13
BCVA (mean logmar +/− SD)	0.35 +/− 0.29	0.23 +/− 0.51	*p* = 0.19
BCVA (mean logmar +/− SD) in the subgroup without comorbidities	0.28 +/− 0.26	0.14 +/− 0.11	*p* = 0.06
Mean induced astigmatism (D +/− SD)	1.72 +/− 0.96	0.72 +/− 0.52	*p* < 0.01
Mean refractive error (D +/− SD)	0.99 +/− 0.57	0.46 +/− 0.36	*p* < 0.01

* Mann–Whitney–Wilcoxon test.

**Table 3:** Summary of postoperative complications.

	Group 1 (Iris Claw) N = 22	Group 2 (STSPFL) N = 20
Macular edema	3 (13.64%)	2 (10.0%)
IOL dislocation	2 (9.09%)	0
Vitreous hemorrhage	1 (4.54%)	1 (5.0%)
Hyphema	1 (4.54%)	0
Neurotrophic ulcer	0	1 (5.0%)
Breakage of the plugs	NA	1 (5.0%)
Conjunctival erosion	0	0
Retinal detachment	0	0

## Data Availability

The data presented in this study are available in the article.

## Supplementary Materials

The following are available online at https://www.mdpi.com/article/10.3390/jcm10102216/s1, Video S1: Carlevale lens fixation.
